# Diagnosis of Autism Spectrum Disorder (ASD) Using Recursive Feature Elimination–Graph Neural Network (RFE–GNN) and Phenotypic Feature Extractor (PFE)

**DOI:** 10.3390/s23249647

**Published:** 2023-12-06

**Authors:** Jiahong Yang, Miaojun Hu, Yao Hu, Zixi Zhang, Jiancheng Zhong

**Affiliations:** College of Information Science and Engineering, Hunan Normal University, Changsha 410081, China; jhyang3668@hunnu.edu.cn (J.Y.); hu@hunnu.edu.cn (M.H.); 202170293825@hunnu.edu.cn (Y.H.); zxzhang@hunnu.edu.cn (Z.Z.)

**Keywords:** autism spectrum disorder (ASD), multimodal, graph neural networks (GNN), ABIDE, functional magnetic resonance imaging (fMRI), recursive feature elimination (RFE)

## Abstract

Autism spectrum disorder (ASD) poses as a multifaceted neurodevelopmental condition, significantly impacting children’s social, behavioral, and communicative capacities. Despite extensive research, the precise etiological origins of ASD remain elusive, with observable connections to brain activity. In this study, we propose a novel framework for ASD detection, extracting the characteristics of functional magnetic resonance imaging (fMRI) data and phenotypic data, respectively. Specifically, we employ recursive feature elimination (RFE) for feature selection of fMRI data and subsequently apply graph neural networks (GNN) to extract informative features from the chosen data. Moreover, we devise a phenotypic feature extractor (PFE) to extract phenotypic features effectively. We then, synergistically fuse the features and validate them on the ABIDE dataset, achieving 78.7% and 80.6% accuracy, respectively, thereby showcasing competitive performance compared to state-of-the-art methods. The proposed framework provides a promising direction for the development of effective diagnostic tools for ASD.

## 1. Introduction

ASD is a neurodevelopmental disorder characterized by social communication impairment, restricted interests, repetitive and stereotyped behaviors, and sensory abnormalities [1]. Treatment for ASD requires substantial financial resources and greatly affects the daily lives of patients and their families. However, diagnosing ASD requires a broad and systematic knowledge of medical practitioners and is also subject to the physician’s subjective factors. Therefore, computer-aided diagnosis (CAD) [2] can provide an effective solution for objective diagnosis.

As fMRI can show changes in blood flow in different areas of the brain, most researchers use fMRI to explore the causes of ASD. Mostafa et al. [3] manually defined some brain features and then used LR, SVM [4], LDA, and KNN to classify fMRI data, achieving 77% accuracy. The manual approach to defining features has significant limitations; it requires knowledge of the relevant domain and the features are limited by the complexity of what can be formulated by humans. Shao et al. [5] and Wang et al. [6] proposed their own methods based on SVM to detect attention deficit hyperactivity disorder (ADHD) and ASD and obtained an accuracy of 77% and 68%, respectively. Methods based on conventional machine learning achieve good classification results on their well-designed feature selection (dimensionality reduction) algorithms. The traditional methods of machine learning cited above can only handle a limited amount of data and cannot yield good results when the amount of data increases. In addition to traditional machine learning, some researchers have also used deep learning algorithms to classify ASD and typical control (TC). Hu et al. [7] proposed a fully connected neural network model to classify ASD patients and TC, and they also explained the features based on the weight of the model. Although a 69.81% accuracy was achieved, the network was designed manually, with a fixed number of model layers and nodes, and limited data, making it difficult to generalize their model. CNN, as the most widely used neural network, has shown its excellent performance in many fields [8,9]. Sherkatghanad et al. [10] directly used their defined CNN model on fMRI data to detect ASD. Even though an accuracy of 70.22% was achieved, the model takes too long to train due to the large number of parameters, and more images need to be used to train a more robust model. In addition, some researchers fed brain correlation matrices and topological features obtained from processed fMRI data into their proposed neural networks and achieved 70.2% accuracy [11]. Their model takes the topological features between each subject and the connections between the features, making it possible to achieve good results while increasing the amount of data the model can handle. It is worth noting that the fMRI data used in these studies are generally sourced from the ABIDE dataset [12]. This dataset increases sample size at the cost of uncontrolled heterogeneity, which causes some disturbances to data samples as well as some loss in classification accuracy.

Brain activity crucially relies on communication between distinct neurons, akin to the node representation update process observed in graph neural networks (GNNs). For the cited research, both traditional machine learning and deep learning methodologies have primarily focused on processing fMRI data to fit their models, e.g., CNN for direct feature extraction on the image and LSTM for the time series data extracted from the fMRI data. This lacks the design of models that effectively simulate inter-neuronal communication within the brain. To address this issue, many investigators use GNN to detect ASD; some investigators have harnessed fMRI data for training GNNs, enabling the simulation of communication patterns among different brain regions through node feature updates [13,14,15]. The potential of including mutual information (MI) loss (Infomax) has been investigated to enable the model to better learn graph embeddings and increase the robustness of the model [13]. They both used graph attention networks to process the input data [14,15]; the former proposed a new method called Pearson correlation-based spatially constrained representation (PSCR), resulting in a modeling accuracy of 72.4%, while the latter enhanced their model’s ability by designing a new attention network and using a larger synthetic graph dataset with 4000 subjects to obtain an accuracy of 68.02%. These methods fit the input patterns of the GNNs by calculating the functional connectivity of the fMRI data. However, these caused a problem: the number of input subjects is small, while the dimensionality of the functional connectivity matrix is large, leading to model overfitting. For this problem, some researchers have used a combination of simple models [16], while others have explored the similarity between samples to make greater use of data [17]. Both of their methods led to some improvement in model accuracy. Many studies have demonstrated the effective capacity of recursive feature elimination (RFE) in the diagnosis of ASD [18,19,20,21]. In their studies, some have used RFE for dimensionality reduction of input data, some have used RFE to reduce the computational complexity of the model, and others have used RFE for feature selection. By comparing the previous studies, they all achieved good results. However, they ignored the contribution of phenotypic data to the classification results. Though several papers incorporate phenotypic data [22,23,24], they predominantly utilize the phenotypic similarity of subjects for graph construction and do not extract features from phenotypic data, relegating it to auxiliary data status within their studies.

To solve the above problems, we propose a novel framework that combines RFE with GNN. Moreover, we explore the correlation between ASD emergence and specific phenotypic data such as age and gender. Our framework segregates the feature extraction process for image data and phenotypic data before unifying them to discern ASD from TC. The key contributions of this paper can be succinctly summarized as follows:

1. There is a limited sample size of medical datasets and a substantial number of feature dimensions in graph data. To overcome this challenge, we leverage RFE to procure a subset of original graph features, which excludes features exhibiting lower classification scores.
2. To combat the issue of overfitting in medical datasets, we incorporate phenotypic data for ASD detection in our research. We devise a novel feature extraction module, termed PFE, which is capable of extracting pertinent features from phenotypic data by continually adjusting its parameters during neural network training, thereby facilitating feature selection. By effectively capturing the underlying representations from phenotypic data, PFE augments the detection of ASD.
3. Leveraging the aforementioned modules, we present a pioneering framework for detecting ASD utilizing multimodal data. Comprehensive experiments conducted on two medical datasets and comparisons with state-of-the-art techniques substantiate the efficacy of the proposed framework. By incorporating RFE and PFE, we surmount the challenge of the high-dimensionality and limited sample size of the datasets while mitigating overfitting. Our framework is endowed with exceptional processing capabilities for multimodal data and can seamlessly integrate diverse modalities to distinguish ASD patients. This has profound implications for doctors to employ computer-aided diagnosis techniques in the prevention and scientific treatment of ASD.

## 2. Materials and Methods

### 2.1. Data Acquisition and Preprocessing

The Autism Brain Imaging Data Exchange I (ABIDE I) serves as a shared repository comprising resting-state fMRI data, anatomical data, and phenotypic data. The extensive complexity arising from the amalgamation of data across 17 distinct data collection sites often leads researchers to test their models on partial datasets. In our investigation, we meticulously excluded incomplete data, focusing on a meticulously selected cohort of 1035 subjects (505 ASD and 530 typical control) to ascertain the efficacy of our proposed framework. Detailed subject information is presented in Table 1. To preprocess the selected image data, we adopted the widely utilized Configurable Pipeline for the Analysis of Connectomes (CPAC).

We used the brain anatomical template cc200 [25] to divide the preprocessed fMRI into 200 ROIs. To transform data into the input mode of the graph neural network, we converted each subject into an undirected graph, G=(V,E,Ω), where $V=\left[v_{1},\ldots ,v_{N}\right]$ denotes nodes set, $E=\left[e_{1},\ldots ,e_{N}\right]$ represents edges set in the graph and is a collection of $\left(v_{i},v_{j}\right)$ linking vertices from $v_{i}$ to $v_{j}$, and $\Omega =\left[\omega_{1},\ldots ,\omega_{N}\right]$ represents the edge weights set. G has an associated node feature set, $H=\left[h_{1},\ldots ,h_{N}\right]$, where $h_{i}$ is the feature vector associated with node $v_{i}$. We use the Pearson correlation coefficient between different ROIs as the node feature in the graph, which is calculated as follows:

(1) $$H=\frac{\sum_{i=1}^{T}\left(\chi_{i}-\bar{\chi }\right)\left(\Upsilon_{i}-\bar{\Upsilon }\right)}{\sqrt{\sum_{i=1}^{T}{\left(\chi_{i}-\bar{\chi }\right)}^{2}}\sqrt{\sum_{i=1}^{T}{\left(\Upsilon_{i}-\bar{\Upsilon }\right)}^{2}}}$$

where χ, Υ represents the time series for different ROI and $\chi_{i}$, $\Upsilon_{i}$ represents the *i*-th time point of χ, Υ, respectively. Based on the Pearson correlation coefficient, we used the absolute value of partial correlation coefficients as the weight of edges to alleviate the over-smoothing.

For phenotypic data, based on previous research [26], we selected the data collection site, sex, age, FIQ, VIQ, PIQ, eye_at_scan, and handedness_category as phenotypic features in this paper. We used one-hot encoding for categorical features. We then normalized the numerical features to the range [0, 1] to eliminate the impact of different scales. Finally, we concatenated the category and numerical features as the final phenotypic feature vector, $P=\left[p_{1},\ldots ,p_{N}\right]$. The procedure can be formulated as Equation (2).

(2) $$p_{i}=O\left(p_{cat}\right)⊕S\left(p_{num}\right)$$

where $p_{i}$ denotes the $i^{th}$ subject phenotypic feature and $p_{cat}$ and $p_{num}$ indicate the category and numerical features vectors, respectively. O(·) and S(·) denote the one-hot encode and scaling, respectively.

For the ABIDE II dataset, the specific information is shown in Table 2. We selected 1110 samples for our study, including 518 ASD and 592 TC. We used the pipeline of Data Processing Assistant for Resting-State fMRI (DPARSF) [27] for preprocessing, along with the website http://rfmri.org/DPARSF (accessed on 25 October 2023), thus showing more detail. Afterwards, as in ABIDE I, we used the cc200 template to partition the brain into 200 ROI to construct the graph structure.

### 2.2. Methods

**Figure 1:**
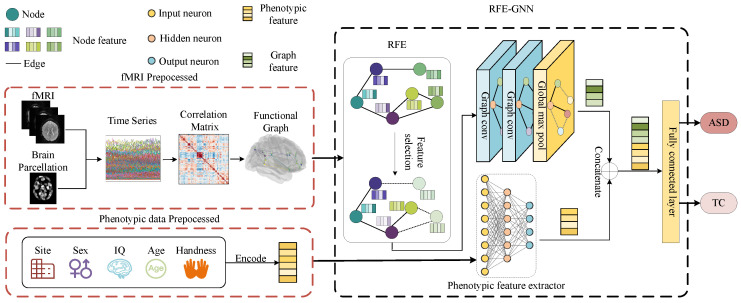
Overview of the RFE–GNN.

This manuscript presents a novel framework, depicted in Figure 1. The image data and phenotype data are respectively preprocessed in different ways to obtain the functional connectivity matrix and vector. Then, they are fed into RFE–GNN for feature extraction, respectively, and finally they are concatenated and fed into the fully connected layer for classification.

#### The Framework of RFE–GNN

Each subject in the original fMRI is preprocessed into a graph, G=(V,E,Ω), and G has an associated node feature set, $H=\left[h_{1},\ldots ,h_{n}\right]$, where *n* is the number of *V*. Since the *H* is a symmetric matrix, and the atlas we used is cc200, the node features for each subject have (19,900(200 × 199 ÷ 2)) dimensions, while the total number of subjects is small, only 1035 in ABIDE I and 1110 in ABIDE II, which causes the typical “small sample, high dimension” problem.

In the context of challenging data scenarios where neural networks struggle to learn meaningful features, often leading to severe overfitting and limited generalization capabilities, we propose employing RFE to mitigate this issue. RFE aids in reducing feature dimensions by leveraging a selected estimator. Subsequently, we utilize the SVM as the estimator to recursively partition the feature space into two distinct parts. The separating hyperplane of the SVM can be formulated using Equation (3).

(3) $$f\left(H\right)=W_{svm}H+b_{SVM}$$

where *H* denotes the node features and $W_{SVM}$ and $b_{SVM}$ are the SVM weights and bias. After each partition, we use the distance between feature points and the separating hyperplane as their importance scores, as formulated by Equation (4):

(4) $$I_{i}=\frac{\left|W_{SVM}h_{i}+b_{SVM}\right|}{{∥W_{SVM}∥}_{2}}$$

where $I_{i}$ denotes the *i*-th importance scores, $h_{i}$ denotes the *i*-th feature, |·| and $\left|\right|\cdot {\left|\right|}_{2}$ denote the l1, l2 norm.

Subsequently, the feature ranking is determined based on the obtained scores; features with lower scores are successively eliminated. This iterative process persists until the number of remaining features matches the predetermined value we have set.

Given that the underlying graph is an undirected weighted graph, and our objective pertains to graph classification, it becomes imperative to aggregate comprehensive global information from the entire graph. To achieve this, we adopt GraphConv [28], which incorporates edge weights while updating node representations. Specifically, GraphConv entails the multiplication of edge weights with neighboring node representations, followed by summation to construct the information of neighboring nodes. Subsequently, this information is propagated to the target node. To mitigate the over-smoothing [29] effect, our proposed framework uses only two layers of GraphConv for learning node embeddings. The specific process can be succinctly represented through the following formula:

(5) $$H_{i}^{l}=W_{1}H_{i}^{l-1}+W_{2}\sum_{j,j\epsilon N_{i}}\left(H_{j}^{l-1}\cdot \Omega_{ji}\right)$$

where $W_{1}$ and $W_{2}$ are network parameters. $H_{i}^{l}$ indicates the *i*-th node feature in the *l*-th layer representation. $N_{i}$ represents the set of neighboring nodes of $v_{i}$, $\Omega_{ji}$ represents the weight coefficient of the edge between $v_{i}$ and $v_{j}$.

Additionally, we integrate BatchNorm layers following each convolution step to accelerate convergence and reduce overfitting. Subsequently, employing global max pooling enables the selection of an appropriate node representation as the overall graph representation. The specific operational process is concisely represented through the following formula:

(6) $$G=max(\left\{h_{1},\ldots ,h_{N}\right\})$$

where G represents the graph representation, *N* represents the total number of nodes in the graph, and $h_{i}$ represents the feature representation of $v_{i}$ in the graph. After graph feature extraction, the data are processed into a two-dimensional vector.

In real-world diagnostic contexts, the reliance on single-modal data alone often proves inadequate for achieving accurate diagnoses. Professional medical practitioners adopt a holistic approach by analyzing multimodal information pertaining to the patient to arrive at comprehensive judgments. Similarly, in the domain of computer-aided diagnosis, we capitalize on the complementarity inherent in diverse modalities of information to make well-rounded assessments. Beyond medical imaging data, non-imaging phenotypic data offers valuable supplementary insights into the associations among subjects. After preprocessing, the phenotypic data are encoded into the vector denoted as *P*. Subsequently, to obtain its latent representation, we design a PFE that comprises an MLP with *L* hidden layers. In this study, we set *L* to 1, and the output vector dimension of the MLP aligns with that of the imaging data features. After processing the original phenotypic data with MLP, its features can be represented as:

(7) $$P^{\prime }=\sigma_{MLP}\left(W_{MLP}P+b_{MLP}\right)$$

where $\sigma_{MLP}$ represents the activation function and $W_{MLP}$ and $b_{MLP}$ represent the layer parameters. *P* and $P^{\prime }$ refer to the original and the processed phenotypic vector, respectively.

Upon extracting features from both image data and phenotypic data, we acquire their respective latent representations. However, directly feeding these representations into the classifier may yield suboptimal results, as the presence of unimportant features can influence the outcomes, stemming from varying importance levels among the data. To address this concern and effectively fuse information from diverse modalities, we introduce an attention mechanism as a viable solution. The attention mechanism efficiently captures and integrates relevant information, thus enhancing the overall performance of the classification process.

In the process of calculating the attention score, we concatenate the representations of image data and phenotypic data as the input *A*, and the operation can be formulated as follows:

(8) $$A=G⊕P^{\prime }$$

where *A* represents the attention input and ⊕ represents vector concatenation.

We use a scaled dot-product model as the scoring function to calculate the weight; therefore, the calculation of the attention coefficient, α, can be expressed in the following formula:

(9) $$\alpha =Softmax(\frac{A^{T}A}{\sqrt{D}})$$

where $A^{T}$ is the transpose of the input vector and *D* represents the dimension of the input vector.

After obtaining the attention score, we multiply it with the original input *A* to obtain the classifier’s input. The classifier consists of two fully connected layers, and each layer is followed by ReLU activation. We chose the cross-entropy function as our objective function, which can be formulated as follows:

(10) $$L=-\frac{1}{K}\sum_{i=1}^{K}y_{i}\log\left({\hat{y}}_{i}\right)$$

where *K* represents the number of subjects and $y_{i}$ and ${\hat{y}}_{i}$ represent the true and predict value, respectively. We minimize the objective function to obtain the best classification performance.

## 3. Experiments and Results

In this section, we meticulously validate the efficacy of our proposed framework in adeptly capturing the salient features present in multimodal data, leveraging the ABIDE datasets. In Section 3.1, we describe the training steps of the model and some parameter settings. In Section 3.2, we present the calculation formulas employed to measure the performance metrics. In Section 3.3, we substantiate the effectiveness of our framework by comparing recent methods. In Section 3.4, the raw features and embedding learned by RFE–GNN over ABIDE datasets were visualization. In Section 3.5, the ablation experiments is used to shedding light on the contribution of individual components. Moreover, ROC curves are plotted, providing a more intuitive visualization of the performance improvements achieved by our framework. In Section 3.6, we scrutinize the impact of diverse hyperparameter values on the overall system performance.

### 3.1. Training Setup

In this study, we implemented our model in the Python environment using the Pytorch library and trained it on the Linux platform using 12 vCPU Intel(R) Xeon(R) Platinum 8255C CPU @ 2.50 GHz with 43 GB memory and an NVIDIA GeForce RTX 2080 Ti with 11 GB GPU memory. The dataset was randomly split into training and test sets in the ratio of 8:2. During the training process, the fMRI data were preprocessed into functional connectivity matrices, after which they were feature-selected using RFE and then fed into GNN to extract features. The phenotype data, on the other hand, were encoded into two-dimensional vectors by one-hot encoding and then feature extraction was performed using PFE. Afterward, we concatenated them and used the attention layer to assign different weights to the features. Finally, the resulting vectors were fed into a fully connected layer to obtain the class to which the subject belonged. The values of the parameters involved in this experiment are shown in Table 3.

### 3.2. Statistical Metrics

As a binary classification task, the commonly used evaluation metrics include Accuracy (ACC), Sensitivity (SEN), Precision (PRE), and F1_score, which can be calculated as follows:

(11) $$\mathrm{ACC}=\frac{\mathrm{TP}+\mathrm{TN}}{\mathrm{TP}+\mathrm{FP}+\mathrm{TN}+\mathrm{FN}}$$

(12) $$\mathrm{SEN}=\mathrm{RECALL}=\frac{\mathrm{TP}}{\mathrm{TP}+\mathrm{FN}}$$

(13) $$\mathrm{PRE}=\frac{\mathrm{TP}}{\mathrm{TP}+\mathrm{FP}}$$

(14) $$F1\_\mathrm{score}=\frac{2*PRE*SEN}{\mathrm{PRE}+\mathrm{SEN}}$$

In the above formulas, TP, FP, TN, and FN represent true positive, false positive, true negative, and false negative, respectively. ACC represents the proportion of correctly predicted samples to the total number of subjects. SEN and RECALL represent the proportion of correctly predicted positive samples to the total positive samples. PRE represents the proportion of true positive samples to the total predicted positive samples. To comprehensively evaluate the model’s prediction performance, we use the F1_score, which considers both PRE and SEN. AUC are also used as an evaluation metric, which is obtained by calculating the area under the ROC curve.

### 3.3. Comparison with State-of-the-Art (SOTA) Models

In this section, we summarize the SOTA methods for detecting ASD, which can be divided into two categories based on the datasets. We also plot the distribution of the accuracy of different methods on different sample sizes. It is worth noting that in our experiments we counted the computational efficiency of our model. Following the setup experiments in Section 3.1, the model training for 100 epochs takes 264.95 s. In the validation mode, it takes only 0.2 s to evaluate a subject.

According to the data presented in Table 4 and Table 5, our framework exhibits superior performance compared to recent SOTA methods. These results substantiate the framework’s ability to effectively extract pertinent features from both image data and phenotypic data.

**Figure 2:**
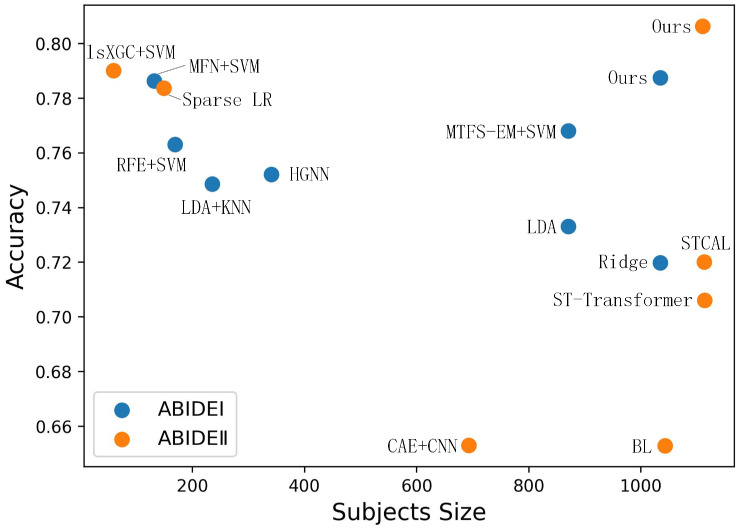
Comparison of the performance with previous literature on ABIDE I and II.

As demonstrated in Figure 2, our framework not only surpasses the performance of most current methods but also successfully handles a larger data sample. This robustness and competitiveness affirm the efficacy of our framework in ASD detection. Furthermore, we observe that for various SOTA methods applied to the ABIDE II dataset, the sample size of their models is considerably smaller than that of ABIDE I. A possible reason for this disparity is the lack of preprocessed data available for the ABIDE II dataset. To address this issue, we utilize preprocessed data derived from the procedural description provided in [39] in our study.

### 3.4. Visualization the Classification

In this section, we use t-SNE [43] to visualize the classification performance of the framework we proposed. t-SNE is a data visualization tool that can reduce high-dimensional data to two dimensions using PCA and represent it on a flat plot.

**Figure 3:**
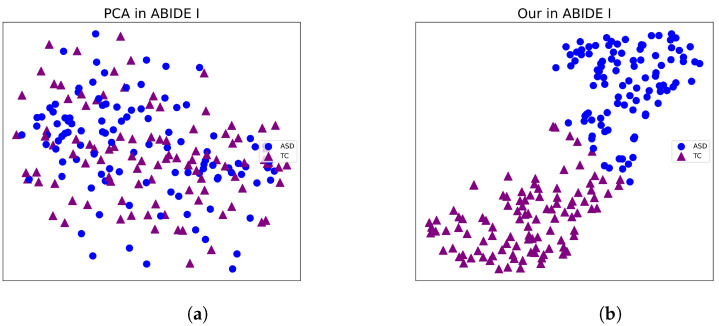
Visualization of the features learned by different methods in ABIDE I. (**a**) PCA in ABIDE I. (**b**) Our in ABIDE I.

**Figure 4:**
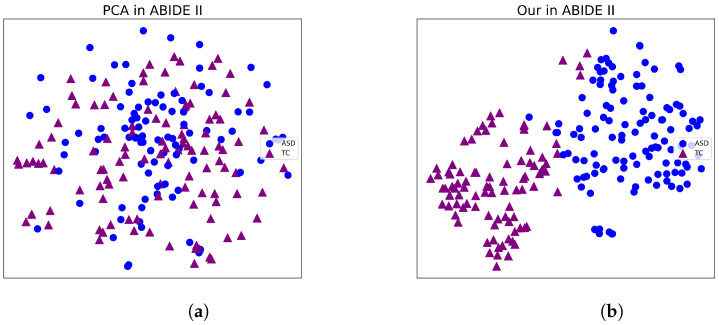
Visualization of the features learned by different methods in ABIDE II. (**a**) PCA in ABIDE II. (**b**) Our in ABIDE II.

Figure 3 and Figure 4 present compelling visualizations of the features learned by our framework. Upon careful examination, it becomes evident that our proposed approach yields superior separability compared to directly reducing the original data input. This observation serves as a strong indication of the efficacy of our proposed framework in effectively extracting relevant features, thereby enhancing the discriminative capabilities of the model.

### 3.5. Comparison with Different Models

In this section, we conduct ablation experiments for different methods, showing the effects of the various modules of our model as well as the robustness of the methods. Furthermore, to provide a visual understanding of the classification performance, ROC curves are adeptly plotted, facilitating insightful observations and conclusive interpretations.

- SVM: SVM finds a hyperplane in the feature space of input data such that the distance between each sample and the hyperplane is maximized, achieving the classification effect of the original data. In the subsequent experiments, we use the SVC model from the sklearn Python library. Parameters are set as regularization parameter = 1.0 and kernel = ‘linear’, and the remaining parameters are set to default.
- RF [44]: Random Forest is an ensemble algorithm that builds multiple decision trees on data and combines the results. The RF model is also found in the sklearn library; the parameter of n_estimators = 1600, and the other is set to default.
- GAT [45]: GAT is a graph convolutional layer based on GCN. It uses a self-attention mechanism to aggregate neighbor nodes and adaptively match the weights of different neighbors. The GATConv we use is in the torch_geometric library, and the layer parameter is set to default; in the GAT model, there are two layers.

From Table 6 and Table 7, we can see that both RFE and PFE contribute to the improvement of model accuracy. When we add both modules to the model, the accuracy is improved even more. Meanwhile, we also use our modules on different models and find that the accuracy is improved to different degrees, indicating that our method is generalizable.

**Figure 5:**
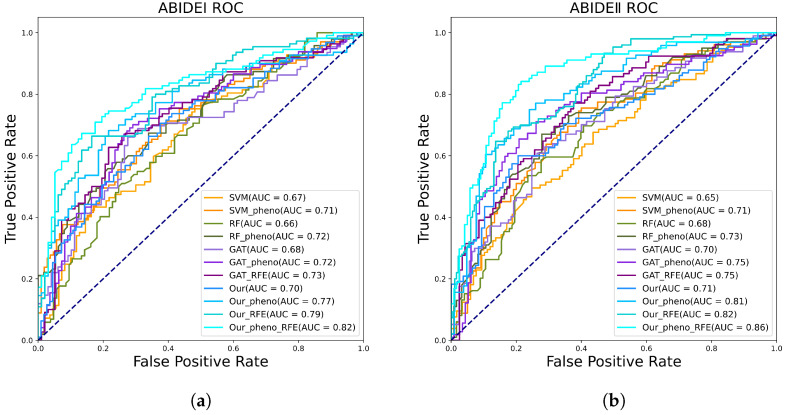
ROC curve comparison of different methods. (**a**) Different methods of ROC on ABIDE I. (**b**) Different methods of ROC on ABIDE II.

As depicted in Figure 5, the AUC serves as a valuable metric denoting the discriminative power of the model. Higher AUC values indicate superior model performance. Our framework achieves AUC values of 0.82 and 0.86 on the ABIDE I and II datasets, respectively, outperforming the comparative methods.

### 3.6. Hyperparameter Discussion

**Figure 6:**
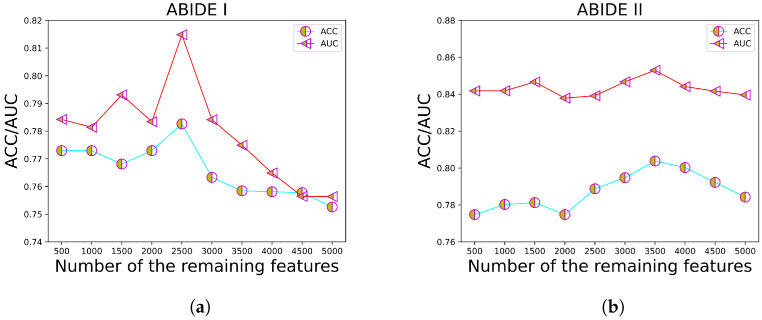
Comparison of different *N* with RFE. (**a**) The impact of our method on ABIDE I. (**b**) The impact of our method on ABIDE II.

To investigate the influence of varying numbers of remaining features when employing RFE, we conduct a thorough examination by adjusting the parameter *N* and observing its impact on dataset performance. Specifically, we set the range of *N* as [500,5000], with increments of 500. We utilize model ACC and the AUC to assess the performance holistically. The results, as depicted in Figure 6, indicate that the ACC and AUC attain their peak values when *N* is set to 2500 for ABIDE I and 3500 for ABIDE II, respectively.

## 4. Discussion

Based on the above results on ABIDE, we validate the effectiveness of the proposed framework. In Section 3.3, by observing the number of subjects, accuracy, and sensitivity, we substantiate our model’s excellent performance. Visualization of the classification demonstrates the discriminability of the features extracted by our framework, which can better classify ASD and TC. These are all due to RFE and PFE. RFE, by leaving behind the graph features that are useful for detecting ASD by recursively ranking the feature of graph data, effectively overcomes the overfitting caused by the large data samples. PFE extracts the phenotypic features through training, which further improves the accuracy. The ablation experiments demonstrate the enhancement of the PFE and RFE and also illustrate that the combination of graph data and phenotypic data to detect ASD is effective and generalizable, which provides a possible method for the CAD of ASD.

There are some limitations in our method. First, in the process of constructing graph data for fMRI, we consider the relationship of spatial scales but ignore the information on temporal scales. Second, in the feature selection for graph data, there are deficiencies in the method of calculating the importance of features. Finally, for the fusion of graph features and phenotypic features, we only perform a simple concatenate of them and do not consider the inter-modal feature relationships. Therefore, in future research, we will explore the above aspects.

## 5. Conclusions

In this paper, we present a novel framework designed for ASD detection utilizing multimodal data. Our approach incorporates RFE to efficiently reduce the number of features fed into the graph convolution layer, mitigating the risk of overfitting. To further enhance performance, we introduce phenotypic data and devise a PFE for effective feature extraction. Through rigorous comparative analysis against recent SOTA methods, we demonstrate the superiority of our proposed framework. The promising results obtained from our research suggest its potential applicability in the realm of CAD, thus contributing to the advancement of ASD detection.

## Figures and Tables

**Table 1:** ABIDE I demographic information.

SITE	ASD						*TC* ^1^					
	Sex (m/f)	Age Avg (SD)	FIQ Avg (SD)	VIQ Avg (SD)	PIQ Avg (SD)	Hand* ^2^ (l/m/r)	Sex (m/f)	Age Avg (SD)	FIQ Avg (SD)	VIQ Avg (SD)	PIQ Avg (SD)	Hand* (l/m/r)
CALTECH	15/4	27.4 (10.3)	107.7 (12.4)	107.3 (15.1)	107 (11.5)	0/5/14	14/4	28 (10.9)	114.8 (9.6)	114.5 (12.8)	111.8 (9.5)	1/3/14
CMU	11/3	26.4 (5.8)	114.5 (11.6)	111.2 (13.7)	114.4 (10.6)	1/1/12	10/3	26.8 (5.7)	114.6 (9.7)	112.8 (9.8)	112.8 (9.8)	0/1/12
KKI	16/4	10 (1.4)	97.9 (17.5)	N/A	N/A	1/3/16	20/8	10 (1.2)	112.1 (9.4)	N/A	N/A	2/3/24
LEUVEN	26/3	17.8 (5)	109.4 (13.1)	99.1 (20)	103.7 (16.8)	3/0/26	29/5	18.2 (5.1)	114.8 (12.9)	116.4 (10.8)	108 (13)	4/1/29
MAX_MUN	21/3	26.1 (14.9)	109.1 (14.1)	N/A	111.5 (10.8)	2/0/22	27/1	24.6 (8.8)	111.8 (9.3)	N/A	110.9 (13.8)	0/0/28
NYU	65/10	14.7 (7.1)	107.1 (16.4)	104.9 (15.9)	108.3 (17.3)	0/0/75	74/26	15.7 (6.2)	113 (13.4)	112.8 (12.7)	110.2 (14)	0/0/100
OHSU	12/0	11.4 (2.2)	105.5 (21.1)	N/A	N/A	1/0/11	14/0	10.1 (1.1)	115 (11.1)	N/A	N/A	0/0/14
OLIN	16/3	16.5 (3.4)	111.3 (17.7)	N/A	N/A	4/0/15	13/2	16.7 (3.6)	113.9 (16.5)	N/A	N/A	2/0/13
PITT	25/4	19 (7.3)	110.2 (14.6)	107 (13.8)	110.8 (14.1)	3/0/26	23/4	18.9 (6.6)	110.1 (9.4)	107.7 (11)	109.6 (9)	1/0/26
SBL	15/0	35 (10.4)	N/A	N/A	N/A	1/0/14	15/0	33.7 (6.6)	N/A	N/A	N/A	0/0/15
SDSU	13/1	14.7 (1.8)	111.4 (18)	110.1 (18.4)	109.7 (16.4)	1/0/13	16/6	14.2 (1.9)	108.1 (10.5)	106.7 (10.4)	107.8 (12.2)	3/0/19
STANFORD	15/4	10 (1.6)	110.7 (16.1)	108.3 (20.4)	110.6 (12.5)	3/1/15	16/4	10 (1.6)	112.1 (15.4)	111.2 (19.7)	110.6 (15.6)	0/2/18
TRINITY	22/0	16.8 (3.2)	108.9 (15.5)	107.9 (14.4)	107.6 (15.7)	0/0/22	25/0	17.1 (3.8)	110.9 (12.2)	109.6 (13.7)	110.3 (10.9)	0/0/25
UCLA	48/6	13 (2.5)	100.4 (13.5)	101.6 (14.1)	99.8 (13.9)	6/0/48	38/6	13 (1.9)	106.4 (11.1)	107.1 (11.6)	104.3 (11.7)	4/0/40
UM	57/9	13.2 (2.4)	105.4 (17.1)	108.7 (20)	102.5 (19.9)	7/1/58	56/18	14.8 (3.6)	107.9 (9.7)	113.6 (12.8)	103 (12)	9/0/65
USM	46/0	23.5 (8.3)	99.7 (16.6)	95 (19.3)	104.7 (16.7)	0/0/46	25/0	21.3 (8.4)	115.4 (15.1)	113.6 (16)	112.8 (14.2)	0/0/25
YALE	20/8	12.7 (3)	94.6 (21.6)	96.5 (23.1)	92.3 (19.2)	6/0/22	20/8	12.7 (2.8)	105 (17.4)	106.8 (16)	101.3 (16.5)	4/0/24

^1^ TC: Typical Control. FIQ: Full Intelligence Quotient. VIQ: Verbal IQ. PIQ: Performance IQ. avg: Average. SD: Standard Deviation. ^2^ Hand*: Handedness.

**Table 2:** ABIDE II demographic information.

SITE	ASD						TC					
	Sex (m/f)	Age Avg (SD)	FIQ Avg (SD)	VIQ Avg (SD)	PIQ Avg (SD)	Hand* ^1^ (l/m/r)	Sex (m/f)	Age Avg (SD)	FIQ Avg (SD)	VIQ Avg (SD)	PIQ Avg (SD)	Hand* (l/m/r)
BNI	29/0	37.4 (16.1)	107.8 (13.7)	N/A	N/A	0/0/29	29/0	39.6 (15.1)	112.4 (12.1)	N/A	N/A	0/0/29
EMC	19/5	8.2 (1.2)	N/A	N/A	99.3 (14.3)	5/0/19	22/5	8.2 (1)	N/A	N/A	99.4 (15.4)	6/0/21
ETH	13/0	20.6 (3.4)	109 (13)	111 (13.3)	105.2 (14.6)	0/0/13	24/0	23.9 (4.5)	116.5 (9.5)	114.2 (14.1)	114.1 (12.4)	0/0/24
GU	43/8	10.9 (1.5)	118.3 (15.4)	120.3 (15.2)	110.7 (15)	8/0/43	28/27	10.4 (1.7)	121.5 (13.8)	121.6 (15.2)	116.5 (13.3)	3/0/52
IP	14/8	15.1 (4.9)	92.4 (24.5)	98.6 (23.3)	92 (22.8)	1/0/21	12/21	23.7 (11.6)	108.1 (18.6)	111.5 (12)	112.9 (11.2)	4/2/27
IU	16/4	25 (9.3)	116.3 (11.8)	117.8 (15)	110.3 (14.4)	2/3/15	15/5	23.8 (4.9)	117 (10.7)	115.3 (10.4)	115 (12.2)	1/2/17
KKI	41/15	10.3 (1.5)	103.4 (16)	109.9 (17.1)	105.3 (14.2)	2/8/46	99/56	10.3 (1.2)	114.3 (10.5)	118 (12.3)	110.7 (12)	10/12/133
KUL	28/0	23.6 (4.8)	106.6 (15.8)	109.6 (11.4)	106.3 (20.7)	6/0/22	N/A	N/A	N/A	N/A	N/A	N/A
NYU_1	43/5	10.1 (5.7)	101.8 (18.3)	101 (16.5)	102.2 (19.1)	2/8/38	28/2	9.5 (3.3)	115.5 (15)	116.1 (15.7)	112.2 (15.2)	0/1/29
NYU_2	24/3	6.8 (1.1)	107.2 (14.1)	110.8 (17.7)	107 (17.2)	4/7/16	N/A	N/A	N/A	N/A	N/A	N/A
OHSU	30/7	11.8 (2.3)	106 (16.7)	N/A	N/A	1/1/35	27/29	10.4 (1.6)	117.5 (12)	N/A	N/A	0/1/55
OILH	20/4	21.8 (3.7)	114 (16.2)	N/A	N/A	4/4/16	20/15	24 (3.6)	111.2 (12.8)	N/A	N/A	0/3/32
SDSU	26/7	12.9 (3.3)	99.8 (14.7)	97.2 (15.5)	103.1 (18.2)	4/2/27	23/2	13.3 (3)	103 (11.7)	104.9 (10.5)	101.4 (14.7)	1/3/21
SU	19/2	11.2 (1.2)	111.8 (15.7)	111.7 (16.8)	109.3 (15.2)	0/0/21	19/2	11 (1.3)	116.1 (14)	117.6 (16.8)	111.4 (13)	0/3/18
TCD	21/0	14.8 (3.3)	108.5 (15.3)	108.5 (15)	106.2 (16.4)	0/0/21	21/0	15.6 (3.1)	118.5 (13.2)	117.3 (15.8)	115.5 (11.8)	0/0/21
U_MIA	11/2	9.9 (2)	100.8 (20.1)	97.5 (21.3)	102.9 (20.9)	0/1/12	11/4	9.7 (2.1)	115.9 (14.7)	112.2 (12.4)	111.7 (19.3)	0/0/15
UCD	14/4	14.8 (2)	103.4 (12.2)	101.1 (15.9)	104.9 (12.8)	0/1/17	10/4	14.8 (1.7)	113 (11.2)	112.1 (10.5)	110.6 (12.8)	0/0/14
UCLA	15/1	11.7 (2.2)	102.1 (14)	101.6 (17.5)	104.4 (15.1)	2/0/14	11/5	9.7 (2.1)	114.5 (13.9)	111.9 (13.6)	114.1 (15.1)	1/1/14
USM	15/2	18.3 (7)	99.3 (20)	99.6 (14.4)	101.1 (17)	0/2/15	13/3	24 (7.8)	115.2 (16.2)	116.1 (14.6)	117.4 (16.4)	0/1/15

^1^ Hand*: Handedness.

**Table 3:** Experimental parameter settings.

Parameter Description	Value
Train epoch	100
BatchSize	64
Learning rate	0.001
Weightdecay	0.05
Stepsize	20
Gamma	0.5
GraphConv layers	2
Optimizers	Adam

**Table 4:** Comparison of the performance of the SOTA methods in ABIDE I.

Methods	Classifier	Samples	Acc	Sen
Yang 2019 [30]	Ridge	505 ASD, 530 HC	0.7198	0.7089
Bernas 2018 [31]	LDA	403 ASD, 468 HC	0.733	0.667
Song 2019 [32]	LDA + KNN	119 ASD, 116 HC	0.7486	0.7167
Madine 2020 [33]	HGNN	155 ASD, 186 HC	0.752	N/A
Jung 2019 [34]	RFE + SVM	86 ASD, 83 HC	0.763	0.792
Liu 2020 [35]	MTFS-EM + SVM	403ASD, 468 HC	0.768	0.725
Zheng 2019 [36]	MFN + SVM	66 ASD, 66 HC	0.7863	0.8
Ours	RFE + GNN + PFE	505 ASD, 530 HC	0.7874	0.7429

**Table 5:** Comparison of the performance of the SOTA methods in ABIDE II.

Methods	Classifier	Samples	Acc	Sen
Liu 2021 [37]	BL	487 ASD, 556 HC	0.6529	0.6293
Zhao 2019 [38]	CAE + CNN	303 ASD, 390 HC	0.653	N/A
Deng 2022 [39]	ST-Transformer	521 ASD, 593 HC	0.7061	0.6875
Liu 2023 [40]	STCAL	521 ASD, 592 HC	0.72	0.744
Zhang 2021 [41]	Sparse LR	60 ASD, 89 HC	0.7836	0.7391
Wismu 2020 [42]	lsXGC + SVM	24 ASD, 35 HC	0.79	N/A
Ours	RFE + GNN + PFE	518 ASD, 592 HC	0.8036	0.7624

**Table 6:** Different method performance in ABIDE I.

Methods	Acc	Sen	Pre	F1_Score
SVM	0.6232	0.5979	0.6077	0.6028
SVM ($\mathrm{pheno}{\;}^{1}$)	0.6522	0.5842	0.6629	0.6211
RF	0.6232	0.5502	0.6579	0.5992
RF (pheno)	0.6667	0.6	0.6477	0.623
GAT	0.6763	0.6972	0.6909	0.6941
GAT (pheno)	0.7005	0.6995	0.7023	0.7009
GAT ($\mathrm{RFE}{\;}^{2}$)	0.715	0.7045	0.7092	0.7068
Ours	0.6715	0.7579	0.6154	0.6792
Ours (pheno)	0.7291	0.7813	0.6944	0.7353
Ours (RFE)	0.7488	0.7579	0.7277	0.7425
Ours (pheno, RFE)	0.7874	0.7429	0.8041	0.7723

^1^ pheno: denotes adding phenotypic information to the model. ^2^ RFE: denotes using recursive feature elimination in the model.

**Table 7:** Different method performance in ABIDE II.

Methods	Acc	Sen	Pre	F1_Score
SVM	0.6216	0.519	0.6567	0.5798
SVM (pheno)	0.6667	0.549	0.6667	0.6022
RF	0.6486	0.5455	0.6207	0.5806
RF (pheno)	0.6712	0.56	0.7077	0.6252
GAT	0.6802	0.6276	0.6477	0.6375
GAT (pheno)	0.7207	0.6542	0.7368	0.6931
GAT (RFE)	0.7252	0.6905	0.745	0.7167
Ours	0.6937	0.6	0.7582	0.6699
Ours (pheno)	0.75	0.7604	0.73	0.7449
Ours (RFE)	0.7808	0.7267	0.7533	0.7398
Ours (pheno, RFE)	0.8063	0.7624	0.8021	0.7817

## Data Availability

In this research, a public dataset was used, which can be found at: https://fcp-indi.s3.amazonaws.com/data/Projects/ accessed on 27 September 2023.

## Abbreviations

The following abbreviations are used in this manuscript:

ASD	Autism Spectrum Disorder
RFE–GNN	Recursive Feature Elimination-Graph Neural Network
PFE	Phenotypic Feature Extractor
fMRI	functional Magnetic Resonance Imaging
ABIDE	Autism Brain Imaging Data Exchange
CAD	Computer-aided Diagnosis
ADHD	Attention Deficit Hyperactivity Disorder
LR	Logistic Regression
SVM	Support Vector Machine
LDA	Linear Discriminant Analysis
KNN	K-nearest neighbors
TC	Typical Control
GNNs	Graph Neural Networks
PSCR	Pearson Correlation-based Spatially Constrained Representation
CPAC	Configurable Pipeline for the Analysis of Connectomes
ROIs	Regions of Interest
FIQ	Full Intelligence Quotient
VIQ	Verbal Intelligence Quotient
PIQ	Performance Intelligence Quotient
MLP	Multilayer Perceptron
ROC	Receiver Operating Characteristic
AUC	Area Under Curve
PCA	Principal Components Analysis
SOTA	state-of-the-art
